# Anterograde transport blockade precedes deficits in retrograde transport in the visual projection of the DBA/2J mouse model of glaucoma

**DOI:** 10.3389/fnins.2014.00290

**Published:** 2014-09-17

**Authors:** Christine M. Dengler-Crish, Matthew A. Smith, Denise M. Inman, Gina N. Wilson, Jesse W. Young, Samuel D. Crish

**Affiliations:** ^1^Department of Pharmaceutical Sciences, Northeast Ohio Medical UniversityRootstown, OH, USA; ^2^Department of Anatomy and Neurobiology, Northeast Ohio Medical UniversityRootstown, OH, USA; ^3^Integrated Pharmaceutical Medicine Graduate Program, Northeast Ohio Medical UniversityRootstown, OH, USA; ^4^Biomedical Sciences Graduate Program, Kent State UniversityKent, OH, USA

**Keywords:** glaucoma, ocular, vision disorders, superior colliculi, optic nerve, neurodegeneration, axonal transport, axonopathy

## Abstract

Axonal transport deficits have been reported as an early pathology in several neurodegenerative disorders, including glaucoma. However, the progression and mechanisms of these deficits are poorly understood. Previous work suggests that anterograde transport is affected earlier and to a larger degree than retrograde transport, yet this has never been examined directly *in vivo*. Using combined anterograde and retrograde tract tracing methods, we examined the time-course of anterograde and retrograde transport deficits in the retinofugal projection in pre-glaucomatous (3 month-old) and glaucomatous (9–13 month old) DBA/2J mice. DBA/2J-*Gpnmb*^+^ mice were used as a control strain and were shown to have similar retinal ganglion cell densities as C57BL/6J control mice—a strain commonly investigated in the field of vision research. Using cholera toxin-B injections into the eye and FluoroGold injections into the superior colliculus (SC), we were able to measure anterograde and retrograde transport in the primary visual projection. In DBA/2J, anterograde transport from the retina to SC was decreased by 69% in the 9–10 month-old age group, while retrograde transport was only reduced by 23% from levels seen in pre-glaucomatous mice. Despite this minor reduction, retrograde transport remained largely intact in these glaucomatous age groups until 13-months of age. These findings indicate that axonal transport deficits occur in semi-functional axons that are still connected to their brain targets. Structural persistence as determined by presence of estrogen-related receptor beta label in the superficial SC was maintained beyond time-points where reductions in retrograde transport occurred, also supporting that transport deficits may be due to physiological or functional abnormalities as opposed to overt structural loss.

## Introduction

The trafficking of molecules and organelles through the extensive network of neuronal cell processes make neurons vulnerable in many disease states. Axons, the major output processes of neurons, often extend great lengths and end in synaptic boutons that exhibit massive metabolic demands, protein turnover, and signaling between the neuron and other cells. Nearly all neuronal proteins are synthesized within the cell body and conveyed along the axon through active axonal transport mechanisms. Conversely, survival factors and proteins for degradation are moved toward the cell body via retrograde transport. Axonal transport is a tightly regulated process performed by molecular motors in the kinesin or dynein family traveling along microtubule tracks. Defects in axonal transport have been reported as some of the earliest pathologies in neurodegenerative disorders (Adalbert et al., 2009; Brady and Morfini, 2010), including glaucoma (Anderson and Hendrickson, 1974; Minckler et al., 1976, 1977; Crish et al., 2010).

Glaucoma is a group of neurodegenerative conditions that together make up the leading cause of irreversible blindness worldwide, estimated to affect 80 million people by 2020 (Quigley and Broman, 2006). Vision loss occurs from retinal ganglion cell (RGC) dysfunction and neurodegeneration. Despite this, the only treatments for glaucoma address a major risk factor, elevated intraocular pressure, rather than the neural changes responsible for vision loss (see Osborne, 2009; Danesh-Meyer, 2011). Despite increased interest, neuroprotective or neurorestorative interventions remain elusive—mainly because of our relatively poor understanding of the progression and mechanisms underlying pathology (McKinnon et al., 2008). Interruption of transport was originally attributed to constriction of the optic nerve head as RGC axons exit the eye (Pease et al., 2000; Quigley et al., 2000), however, more recent work suggests subtler intra-axonal mechanisms play a major role in these pathologies (Crish et al., 2010; review in Crish and Calkins, 2011). It has been suggested that anterograde transport deficits precede retrograde transport deficits in the progression of glaucomatous pathology (Buckingham et al., 2008; Crish et al., 2010), but these deficits have never been directly compared. Furthermore, it is unknown how much of the retrograde transport deficits are due to axon/terminal loss rather than dysregulation of axonal transport machinery or similar issues. The precedence of anterograde transport deficits suggests that there are specific, novel mechanisms related to cytoskeletal alterations or molecular motors underlying the pathogenesis of glaucoma. Most importantly, this indicates that an intact, semi-functional axon persists after the onset of pathology—opening up new avenues of potential therapies focused on restoring or protecting neuronal function rather than replacing lost structure.

Using anterograde and retrograde tract tracing methods, we examined the time-course of transport deficits in the retinofugal projection in the DBA/2J mouse model of glaucoma. Anterograde transport deficits were pronounced at 9–10 months of age, whereas retrograde transport from the superior colliculus (SC) to the retina remained largely intact until 13 months of age. Structural persistence of the SC was maintained beyond time-points where reductions in retrograde transport occurred, indicating that transport deficits are likely due to physiological or functional abnormalities as opposed to structural loss.

## Material and methods

### Animals

This study used mixed-sex DBA/2J (*n* = 27; 6–9 per age group), DBA/2J-*Gpnmb*^+^ (*n* = 15), and C57BL/6J (*n* = 7) mice of different ages. The DBA/2J mouse has two loss of function mutations that produce iris atrophy resulting in age-related elevation of intraocular pressure (IOP) and a course of degeneration of visual structures (John et al., 1998). Together, these characteristics resemble ocular hypertension-induced structural changes to the eye as seen in some forms of human glaucoma. DBA/2J-*Gpnmb*^+^ mice have the same background as the DBA/2J, however, they express a functioning wild type *Gpnmb*^+^ allele that prevents them from developing elevated IOP or glaucomatous pathology (Howell et al., 2007). Given the recent development of DBA/2J-*Gpnmb*^+^ mice as controls for glaucoma (Howell et al., 2007; Porciatti et al., 2010), we also used C57BL/6J mice as a comparison since the C57 strain has been commonly used as a control strain in the visual system (Crish et al., 2010; Porciatti et al., 2010). All animals were originally obtained from The Jackson Laboratory (Bar Harbor, ME) and then housed and aged in the Comparative Medicine Unit at Northeast Ohio Medical University to the following time points. For the DBA/2J model, we used 3 month old mice representing the pre-glaucomatous ages, 9–10 month old mice representing ages at the onset of glaucomatous pathology where anterograde transport deficits and mild axonopathy are evident, 11–12 month old mice representing increasing transport deficits and axonopathy, and 13 months where retrograde transport deficits along with axon and cell body loss is evident (Buckingham et al., 2008; Crish et al., 2010). For controls, we used C57BL/6J mice from a convenience sample that ranged in age from 4–17 months, and 9–13 month old DBA/2J-*Gpnmb*^+^ mice—ages targeted for representing the onset of significant glaucomatous pathology in the DBA/2J mice. Mice were maintained in a 12 h light/dark cycle with standard rodent chow available *ad libitum*. All mice were housed in the same room under the same environmental conditions. The Northeast Ohio Medical University Institutional Animal Care and Use Committee approved all experimental procedures.

### Anterograde transport labeling (intravitreal injections)

Mice were placed prone in a stereotaxic device (Stoelting, Wood Dale, IL) equipped with a nose cone set to deliver 2.5% isoflurane at 0.8 ml/min. Injections (1.5 μ l) of 1% cholera toxin subunit B (CTB) conjugated to Alexa Fluor-488 (Invitrogen, Carlsbad CA) in sterile phosphate buffered saline (PBS) were administered into the vitreal chamber of each eye using a 33-ga needle attached to a 25 μ l Hamilton syringe. Animals remained anesthetized in the stereotaxic device for immediate surgical preparation for the retrograde labeling procedure. CTB was used because it is selectively taken up by RGCs (except in the cases of advanced retinal pathology; Crish et al., 2010; Inman et al., 2012)—allowing us to determine uptake and transport in the retina. Furthermore, CTB has significant advantages over other commonly used tracers for anterograde transport in that it travels along all axons of the retinofugal projection and is visible in each component (Angelucci et al., 1996).

### Retrograde transport labeling (intracranial injections)

Surgical sites on the dorsal surface of the skull for mice were cleaned and prepared for the craniotomy procedure. Craniotomies (each 1 mm^2^) to expose the surface of the brain were drilled into each side of the skull using a 0.5 mm burr attached to a micro-motor drill. Two injection sites per hemisphere were used to administer FluoroGold to the entire rostro-caudal span of the SC and were delivered at the following stereotaxic coordinates (each relative to bregma and sagittal suture skull landmarks): −4.0 mm anterior-posterior, ±0.5 mm medial lateral; and −4.4 mm anterior-posterior, ±0.5 mm medial lateral. At each craniotomy site, a 25 μ l Hamilton syringe equipped with a 33-ga needle was lowered (via stereotaxic device) at a 45° angle to a depth of 0.5 mm below the skull surface. This approach differs from previous studies (e.g., Buckingham et al., 2008) in that it minimizes damage to the injection site within the SC, allowing analysis of intact anterograde transport from the retina and RGC marker labeling to determine structural persistence. Once at injection depth, 1 μ l of 3% FluoroGold (FG) (Fluoro-Gold, Fluorochrome; Denver, CO) in 10% dimethyl sulfoxide (DMSO)/PBS was injected. After each injection was complete, the needle was retracted over 30 s. Sterile bone wax was used to seal each of the skull openings and the incision site was closed with tissue adhesive (Vetbond; 3M, USA). Animals were allowed to recover for 72 h before sacrifice. FG was selected as our retrograde tracer because it is the most commonly used tracer for retrograde labeling of RGCs and it is taken up and packaged passively (Wessendorf, 1991). As long as RGC structure is present, FG is able to get inside the cell and into its vesicles. Any lack of FG-positive cell bodies seen in an intact projection would be due to issues such as transport blockade or similar deficits.

### Tissue collection and preparation

Seventy-two hours after injections, subjects received a single intraperitoneal injection of 120 mg/kg sodium pentobarbital and were transcardially perfused with 4% paraformaldehyde in PBS solution. This time frame was selected to allow both tracer applications to be performed at the same time—longer survival times typically used for FG tracing have been reported to result in loss of anterograde tracer in the retinofugal projection in a similarly sized mammal (Kahn and Krubitzer, 2002). While retrograde transport is slower than the fast anterograde transport CTB uses, this is still an adequate amount of time to cover the distances needed. The brains, optic nerve, and eyes were harvested and retinas were dissected from the eyes. Before histological preparation, the cortex overlying SC was removed and the brain was photographed to determine FG placement. Failed FG injections were identified by lack of tracer coverage of the majority of SC surface and these projections were then excluded from analysis (Abbott et al., 2014). Failed CTB injections were identified by lack of tracer in the retina and these projections were also excluded from analysis. Retinas were prepared as flattened whole-mounts: four radial cuts were made in the surface, vitreous humor was removed, and retinas were mounted nerve fiber layer side up on slides. Slides were then covered with Fluoromount-G mounting media (Southern Biotech; Birmingham, AL) and cover-slipped.

### Immunohistochemistry for structural markers

In order to assess whether axon loss was driving deficiencies in transport from the SC, we stained for estrogen-related receptor beta (ERRβ) which labels all components of retinal ganglion cells including their projection to the SC (Real et al., 2008; Crish et al., 2010). Fifty μm coronal serial slices through the SC were taken using a freezing sliding microtome. Every third section of the serial SC slices were added to a 96-well plate containing 200 μ l (per well) block solution (5% donkey serum, 1% Triton-X 100 in 1× PBS) for 2 h at room temperature. Sections were then incubated in primary antibody solution containing rabbit polyclonal antibody ERRβ (1:500, Sigma-Aldrich, E0156; St. Louis, MO) in 3% donkey serum, 1% Triton-X 100 in PBS for 72 h at 4°C, removed and washed 3× for 10 min, and then incubated in Alexa Fluor-594 conjugated secondary antibody (1:200, JacksonImmunoresearch; West Grove, PA) overnight at 4°C. Sections were rinsed 3 × 10 min in PBS before being mounted on microscope slides, and cover slipped with Fluoromount-G. Twenty μm sections of optic nerves were taken parallel to their long axis and stained for the axonal cytoskeletal proteins β-Tubulin (Covance, PRB-425P, 1:5000; Greenfield, IN) and superphosphorylated heavy chain neurofilament (pNF, Covance, SMI-310; Greenfield, IN). Secondary antibodies used for these assays were donkey anti-rabbit Alexa Fluor-594 conjugated (for β-Tubulin) or donkey anti-mouse Alexa Fluor-647 conjugated (for pNF; Jackson ImmunoResearch, West Grove, PA).

### Microscopy

Retina and sections containing SC were photographed with a Zeiss AxioZoom V16 epifluorescent microscope equipped with a digital high-resolution camera (AxioCam MRm Rev.3; Zeiss, Jena, Germany) and a computer guided motorized Z and X-Y stage. Retinal whole-mount reconstructions were obtained by multi-frame acquisitions captured side-by-side with 10% overlap with a 3.6×/0.50 objective (Plan-Neofluar, Zeiss, Jena, Germany) at a final magnification of 160×. Each frame was Z-stacked at an interval of 4–8 μm, producing a stack between 5–10 images. Multi-frame tiles were defined to cover the whole retina completely. Tiled images were aligned to eliminate overlap and create a montage of the entire retinal surface using stitching software in Zen Pro (Zeiss; Jena, Germany). Superior colliculi from each animal were imaged at 80× under multiple channels to capture label from CTB and ERRβ. Optic nerves were imaged on an Olympus FV-1000 confocal microscope (Olympus, Cedar Valley, PA).

### Measurement of retrograde transport

For analysis, Z-stack retina images were flattened into a single layer TIFF using extended depth of focus (EDF) software in Zen Pro. ImagePro (Media Cybernetics; Rockville, MD) software was used to count FG-labeled RGCs in the retina using subroutines similar to ones published by Salinas-Navarro et al. (2009a). In brief, we applied a sequence of 2-D filters to each retinal image that (a) defined and separated individual cells and (b) established criteria for cell size boundaries and roundness and (c) removed artifacts/noise. In order to calculate density of FG-labeled RGCs in each retina, we used the ImagePro software to measure the area of each retina. We divided the total number of FG-positive RGCs counted in each retina by the total area of that retina to yield a density measure of cells/mm^2^. To quantify sectorial loss of FG labeling in retina characteristic of glaucomatous pathology, we measured the area of sections within the retina where FG-positive cells were absent. We subtracted these areas from the total area of the retina to produce a value that represented total area of intact FG and then divided the total area of intact FG by the total retinal area to create the variable “percent area intact” for analysis of intact retrograde transport. This variable was created to allow direct comparison with deficits in the retinotopic collicular map.

### Measurement of anterograde transport and structure

We quantified CTB and ERRβ signal density in multiple slices of the SC using a custom-written macro for NIH ImageJ (Rasband, 1997–2014). This macro performed the same function as the techniques described in Crish et al. (2010) for quantifying signal density in the SC. We set background intensity for each SC section by selecting a region of nonretinorecipient SC (layers IV-VII) or periaqueductal gray for comparison with the retinorecipient SC. We outlined the retinorecipient layers of the SC using layer IV as the ventral border and created bins of pixels running from medial to lateral SC. The number of pixels within bins with CTB or ERRβ signal brighter than background were divided by the total number of pixels in the bin to provide either CTB or ERRβ signal density at that location. We were able to visualize anterograde transport deficits and structural loss in the collicular retinotopic map by constructing colorimetric representations of CTB or ERRβ density [ranging from 0% (blue) to 100% (red)] at each mediolateral location in the SC section and aligning them using Origin Pro software (OriginLab; Northampton, MA). The methods used to create these representations are adapted from Crish et al. (2010) and the code for the ImageJ macro is freely available upon request from the authors.

### Statistical analysis and variables

We used two-way between-subjects factorial analyses of variance (ANOVA) with *post-hoc* Fisher's Least Significant Difference (LSD) tests to determine differences in transport between strains/ages. To compare differences in magnitude between anterograde, retrograde, and structural label within subjects for each age group, we used paired *t*-tests to delineate the effects of these planned comparisons. We also used one-way ANOVA to assess control data and Pearson's correlations for additional descriptive measures. Retrograde transport was defined as either FG-positive cell density in retina or percent area of intact FG staining in retina. Anterograde transport was defined as percent area fraction of intact CTB label in the SC contralateral to the retina analyzed for retrograde transport. Additionally, the structural intactness of each SC was measured by percent area fraction of intact ERRβ label. Retrograde and anterograde transport were compared within the same projection. Each retina and corresponding contralateral SC set were analyzed separately within each animal, as glaucomatous pathology differentially affects each eye thus necessitating the analysis of each projection as an independent measure (Schlamp et al., 2006; Crish et al., 2010).

## Results

### FG density does not differ by strain or age in C57BL/6J and DBA/2J *Gpnmb*^+^ control mice

**Figure 1:**
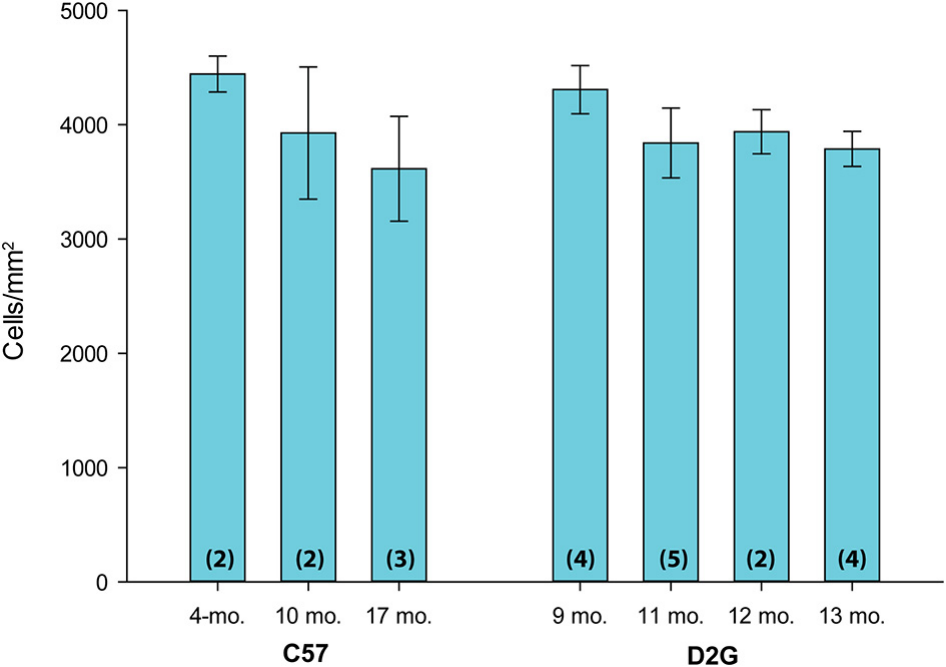
**FluoroGold (FG) density in the retina across age and control strains**. Mean FG-positive cell density (cells/mm^2^) in the retina is shown on the y-axis; age and strain are shown on the x-axis. C57BL/6J (C57) ranged in age from 4 to 17 months old (mo.). DBA/2J *Gpnmb*^+^ (D2G) mice ranged in age from 9–13 mo. There were no significant differences in mean FG density in the retina between strains or ages [ANOVA: *F*_(6, 22)_ = 1.18, *P* = 0.365, *ns*]. Number of cases per group are shown in parentheses. Error bars show standard error of the mean for each group.

FG-positive cell density in retina from C57BL/6J (C57) and DBA/2J-*Gpnmb* (D2G) control strains were compared across age groups to determine if the FG distribution pattern in D2G mice matched that of C57s and if there was similarity between younger and older controls. We compared the density of FG-positive cells in the retina across C57 mice (age range 4–17 months) and D2G mice (age range 9–13 months). Results of an ANOVA indicated no significant differences between ages or strains, *F*_(6, 22)_ = 1.18, *P* = 0.365, *ns* (Figure 1). This validated our use of D2G control mice as a comparable (and more background relevant) substitute for the C57 control strain. Additionally, as these numbers are similar to other studies using FG to label RGCs (e.g., Buckingham et al., 2008), this validated our modified method of FG application. Furthermore, this is the first reported analysis of FG transport in D2G mice. For the remaining analyses, we pooled the D2G control data across ages where we typically see glaucomatous pathology in the DBA/2J (9–12 months old) to form a single control group for this strain.

### Major findings

**Figure 2:**
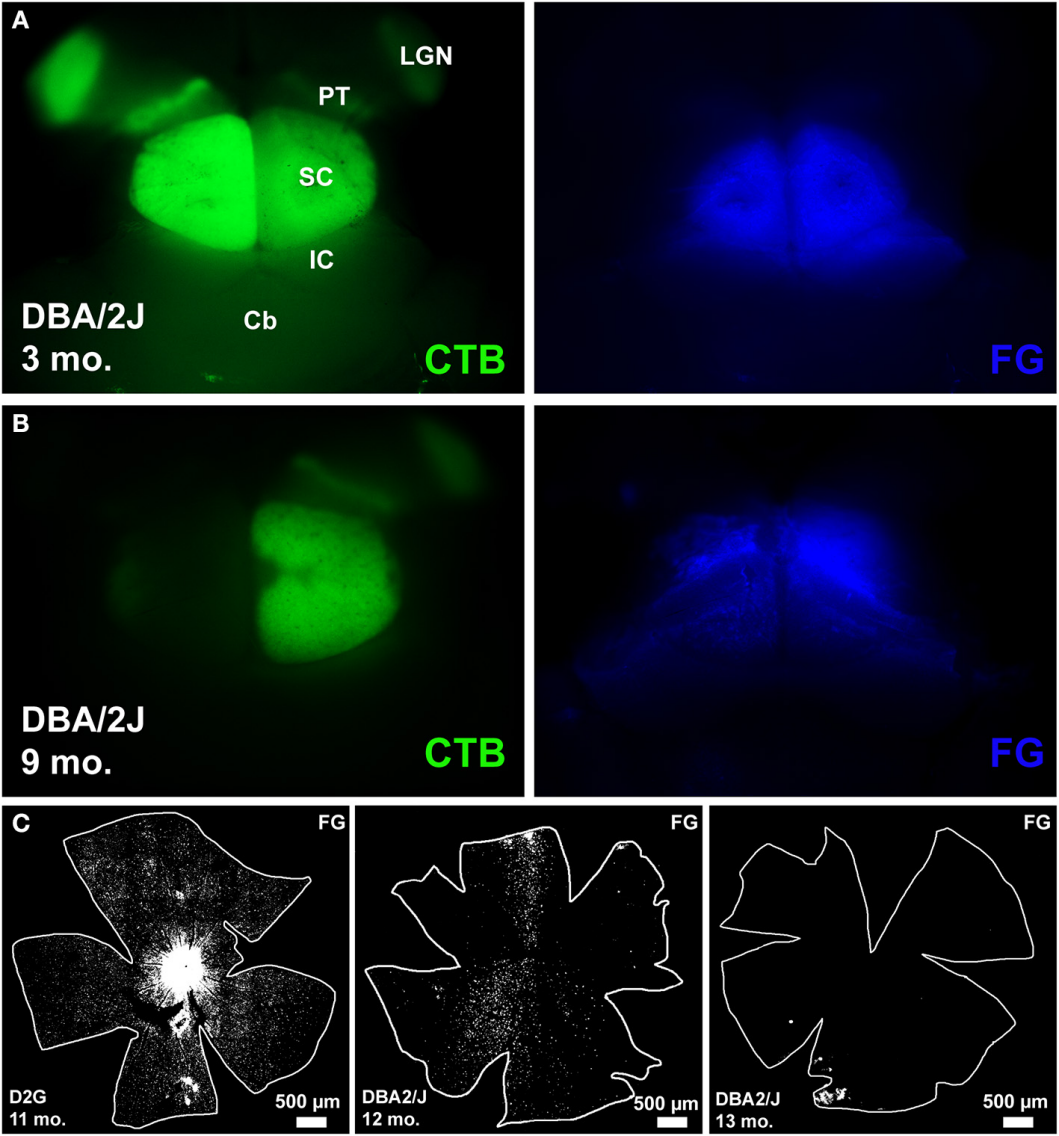
**Examples of tracing in whole mount brain (cortex removed) and retina**. **(A)** left: shows clear anterogradely-transported CTB label in the major retinal targets that include lateral geniculate nucleus (LGN), pretectum (PT), and superior colliculus (SC), other identifying landmarks outside the visual pathway include inferior colliculus (IC) and cerebellum (Cb). **(A)** right: shows good coverage of the SC with retrograde tracer FluoroGold (FG). **(B)** left: shows a near complete loss of CTB in the left SC and nearly no loss in the right SC. **(B)** right: FG covers the SC completely. **(C)** Illustrates the range of retrograde transport of FG we found, from complete coverage in the leftmost panel, to sectorial loss in the middle panel, and a near complete loss in the rightmost panel.

The following abbreviations will be used to label the DBA/2J age groups represented by our data: D3 (3-month old DBA/2J), D9-10 (9–10 month old DBA/2J), D11-12 (11–12 month old DBA/2J), and D13 (13-month DBA/2J). Figure 2 shows examples of tracing in whole mount brain (cortex removed) and retina. The left panel in row A shows clear anterogradely-transported CTB label in the major retinal targets (lateral geniculate nucleus, pretectum, and SC) and the right panel in row A shows good coverage of the SC with FG. Row B (left) shows a near complete loss of CTB in the left SC and nearly no loss on the right (right panel, row B). FG covers the SC completely. Row C illustrates the range of retrograde transport of FG we found, from complete coverage in the leftmost panel, to sectorial loss in the middle panel, and a near complete loss in the rightmost panel.

**Figure 3:**
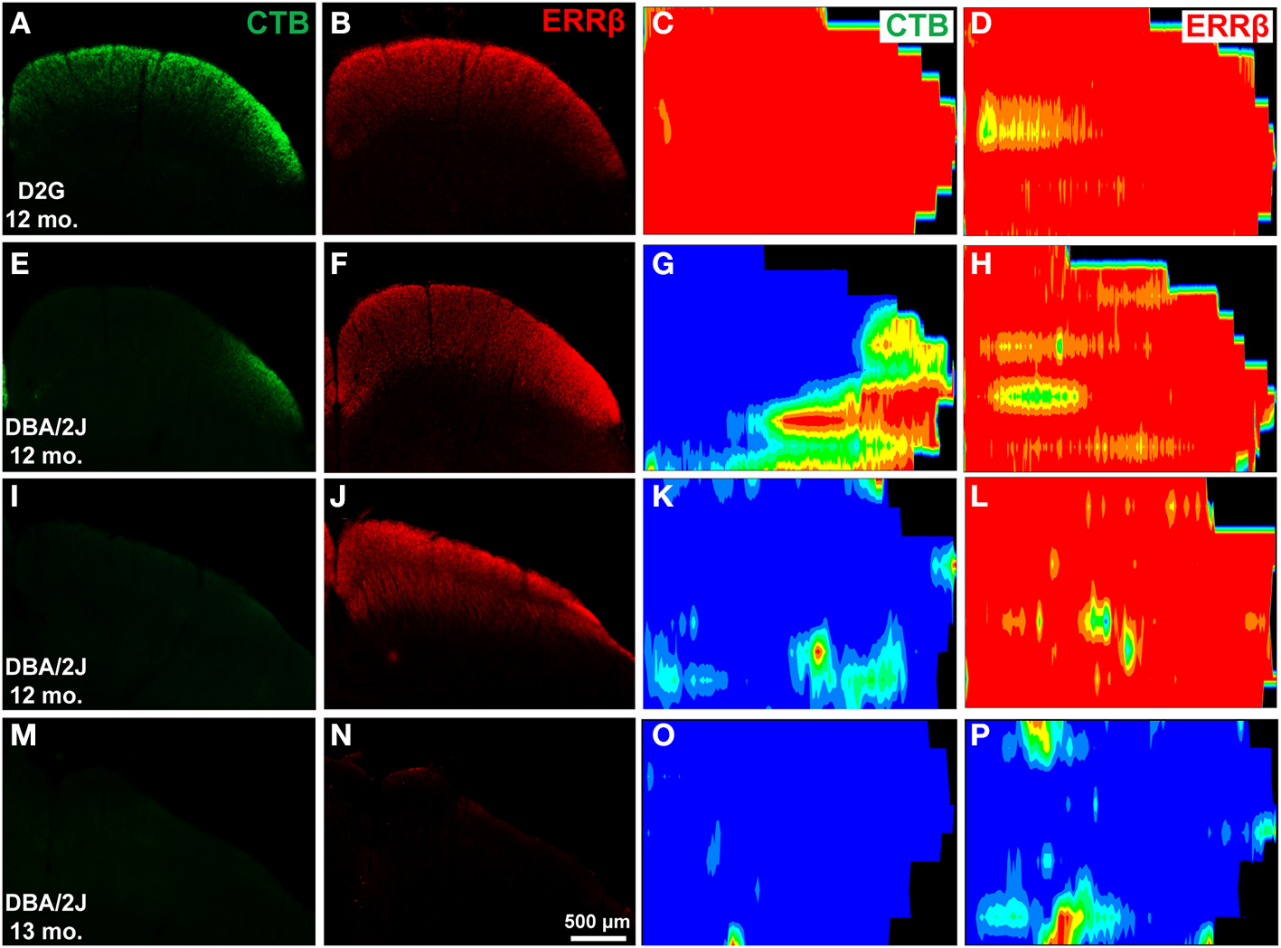
**Differential transport loss with structural persistence in the superior colliculus (SC) of mice**. The first two columns are cross-sections through the midbrain showing the superior colliculus. **(A,B)** Robust CTB and ERRβ label were evident in superficial SC of a control D2G mouse at 12 months of age. **(C,D)** are colorimetric reconstructions of the entire SC, warmer colors are denser with red representing 100% of the pixels at that location positive for either CTB **(C)** or ERRβ **(D)**. As expected, this colliculus demonstrates a uniformly dense labeling across the entire SC. In aged DBA/2J mice, reductions in CTB label were often found (**E,I**; **G,K**). These deficits occurred in sectorial patterns, with some areas devoid of CTB (represented by blue) and some with strong label (right side of **G**). In DBA/2J mice up to 12 months of age, retinal ganglion cell (RGC) structural marker ERRβ appeared normal even in colliculi with severe CTB loss (**F,J**; **H,L**). In 13-month DBA/2J mice we saw significant loss of ERRβ label **(N,P)** along with absence of CTB label **(M,O)**.

In D2G (Figures 3A,B) and young DBA/2J (D3) mice (data not shown), uniform CTB and ERRβ were evident in superficial SC. Colorimetric reconstructions of the entire SC demonstrated a uniformly dense labeling (indicated by red/orange/yellow colors) across the entire SC (Figures 3C,D). In aged DBA/2J mice, reductions in CTB label were often found (Figures 3E,I,M,O). Deficits in the SC occurred in sectorial patterns, with some areas devoid of CTB (indicated by blue/green color) and some with strong label (Figure 3G). In DBA/2J mice up to 12 months of age, RGC structural marker ERRβ appeared normal even in colliculi with severe CTB loss (compare Figures 3F,H with E,G; also J,L with I,K). In 13-month DBA/2J mice we saw significant loss of ERRβ label as well (Figures 3N,P).

**Figure 4:**
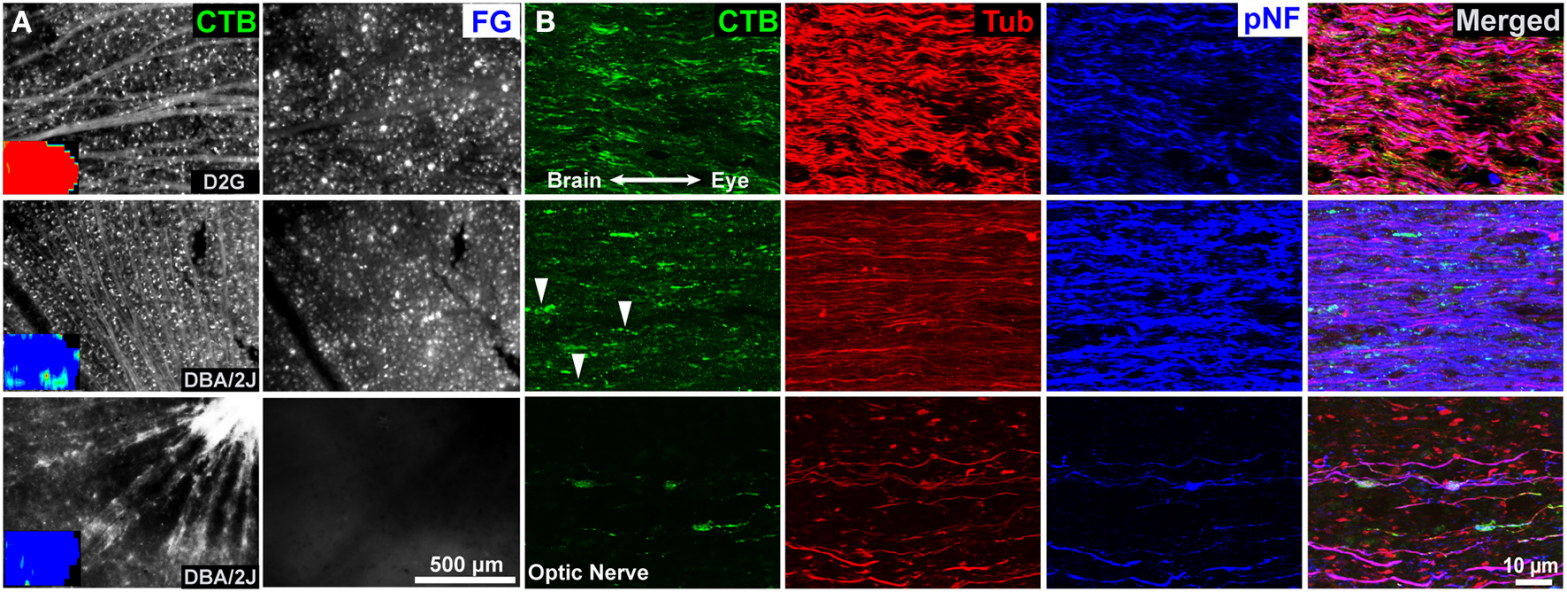
**Retina and optic nerve of three 12-month mice**. **(A)** Representative images of whole mount retina showing CTB uptake and transport in retinal ganglion cells (RGCs). Insets are collicular CTB maps; red represents 100% of the pixels at that location positive for CTB, blue is no label. Top row: Control D2G mice with normal anterograde and retrograde transport. Dense CTB label is evident in RGC somata and axons, and retrogradely FluoroGold (FG) labeled RGCs are readily visible. Middle row: 12-month old DBA/2J with significant loss of CTB but ERRβ persistence. Despite a near complete loss of anterograde transport of CTB to the superior colliculus (inset), RGCs in the corresponding retina still take up and transport the CTB and FG tracers. Bottom row: 12-month old DBA/2J with advanced pathology—complete loss of both CTB and ERRβ in the SC. RGCs are still present and taking up/ transporting CTB within the retina. There is a loss of retrogradely transported FluoroGold (FG) in the retina. **(B)** Corresponding optic nerves near the optic chiasm. CTB is readily apparent in axons even in animals lacking collicular CTB (middle and bottom rows). The second row contains some “string of pearls” varicosities indicative of intra-axonal transport blockades as indicated by arrowheads. The projection with the most severe pathology (bottom row) contains large end-bulbs indicative of dying-back or distal Wallerian degeneration. β-Tubulin (Tub) labeling was lower in the DBA/2J mice with anterograde transport deficits (middle and bottom rows). Superphosphorylated heavy-chain neurofilament (pNF) labeling was increased in animals with selective anterograde transport deficits (middle row) and reduced in animals with severe deficits in both forms of transport (bottom row).

As seen previously (e.g., Crish et al., 2010), lack of CTB label in the SC is not due to loss of RGC somata or axons. CTB-positive RGCs and axons are readily visible in whole mount retina—even in projections with complete absence of collicular CTB (Figure 4, first column). Dense concentrations of FG-positive RGC somata (FG is not typically visible in axons) are also obvious in all but some of the oldest animals studied here (Figure 4, second column). Examination of the optic nerves show persistence of CTB-positive axons in the distal-most portions of this structure (Figure 4, third column). Interestingly, these axons appear to have altered levels of cytoskeletal proteins. In contrast to D2G control mice, β-tubulin levels in the optic nerve of DBA/2J mice appear reduced whereas pNF is increased in mice with significant transport deficits (Figure 4, Tub & pNF columns). At advanced pathological stages where ERRβ loss is seen, β-tubulin and pNF are substantially reduced, indicating axon degeneration (Figure 4, bottom row).

In order to quantitatively compare intact retrograde transport (FG), anterograde transport (CTB), and structure (ERRβ) for each group of mice as well as compare how these values changed as a function of age in the DBA/2J mice, we used a 3 (CTB, FG, ERRβ) by 5 (D2G, D3, D9-10, D11-12, D13) factorial ANOVA for analysis. Significant main effects for label [*F*_(2, 104)_ = 29.98, *P* < 0.001] and strain/age [*F*_(4, 104)_ = 91.57, *P* < 0.001] were shown. A significant interaction between type of label and strain/age group qualified these main effects [*F*_(8, 104)_ = 8.64, *P* < 0.001]. *Post-hoc* Fisher's LSD tests were used to define the significant pairwise comparisons as described below.

### Anterograde, retrograde, and structural label do not differ between D2G controls and 3-month old DBA/2J mice

**Figure 5:**
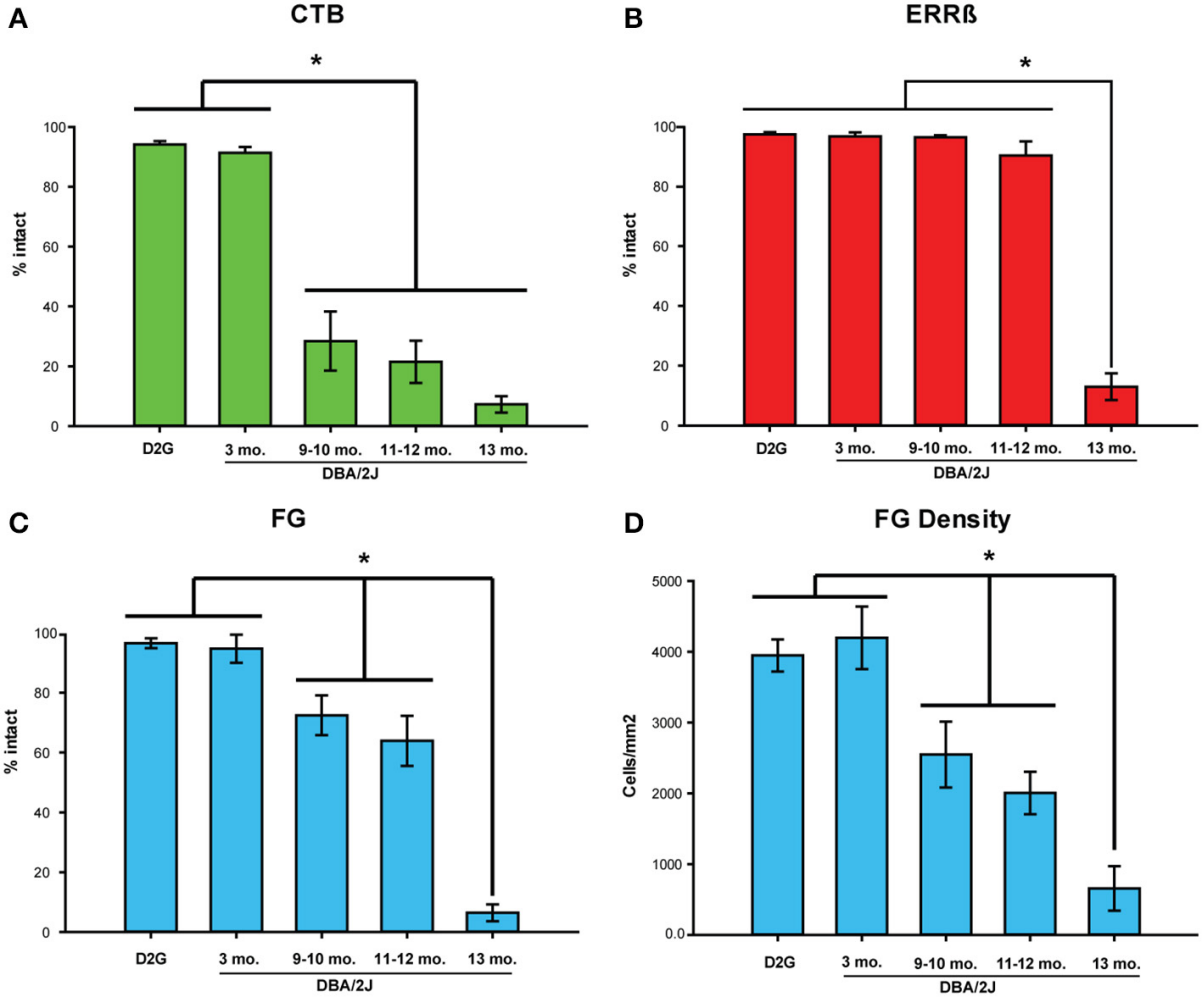
**Intact label across strains/age groups**. **(A)** Mean percent area fraction of intact CTB (anterograde) label and **(B)** ERRβ (axon terminal) label in the superior colliculus (SC). **(C)** Mean percent area of intact FluoroGold (FG; retrograde) label in the retina. No significant differences in any of the three labels were shown between DBA/2J*-Gpnmb*^+^ (D2G) control and 3-month old (mo.) DBA/2J mice (CTB: *P* = 0.808, *ns*; ERRβ: *P* = 0.943, *ns*; FG: *P* = 0.831, *ns*). **(A)** CTB was significantly reduced in 9–10 mo., 11–12 mo., and 13 mo. animals compared to 3-mo. DBA/2J (P < 0.001 in all cases). **(B)** Significant reduction in ERRβ label is only seen in 13-mo. DBA/2J mice. **(C)** FG label was significantly reduced in 9–10 mo. (*P* = 0.02) and 11–12 mo. (*P* < 0.001) mice compared to 3-mo. DBA/2J. FG label in the D13 group decreased significantly from the 11–12 mo. group (*P* < 0.001). **(D)** Mean density (cells/mm^2^) of FG-positive cells in the retina. Results of analyses using FG density variable recapitulate results using percent intact variable shown in **(C)**. Mean FG density and percent area intact FG variables are correlated, *r* = 0.80, *P* < 0.001. The number of cases per group are as follows: D2G = 8; DBA/2J 3-mo. = 6, 9-mo. = 6, 11–12 mo. = 9, and 13-mo. = 6. Asterisks and brackets indicate statistically significant differences between specific groups. Error bars show standard error of the mean for each group depicted.

As anticipated, there were no significant differences in intact anterograde (*P* = 0.808, *ns*), retrograde (*P* = 0.8310, *ns*), or structural label (*P* = 0.943, *ns*) between D2G controls and D3 (pre-glaucomatous) mice (Figures 5A–C). Therefore, we only report *post-hoc* pairwise comparisons between the DBA/2J age groups.

### Anterograde transport (CTB) in SC is significantly reduced at 9-months of age in DBA/2J mice

Glaucomatous DBA/2J (D9 and older groups) showed a marked reduction in CTB label in the SC compared to the D3 group (Figure 5A). The D3 group had significantly more intact label than D9-10, D11-12, and D13 (Fisher's LSD *P* < 0.001 in all three comparisons). Fisher's LSD tests indicated that there were no differences in CTB label between the three oldest DBA/2J groups (D9-10 vs. D11-12: *P* = 0.545, *ns*; D9-10 vs. D13: *P* = 0.098, *ns*; D11-12 vs. D13: *P* = 0.219, *ns*).

### Structure of SC (ERRβ) remains intact until 13 months of age in DBA/2J

The persistence of RGC axon terminals (percent intact ERRβ) in the SC did not differ between D3 and D9-10 (Fishers LSD; *P* = 0.978, *ns*), D3 and D11-12 groups (*P* = 0.088, *ns*), or between D9-10 and D11-12 groups (*P* = 0.094, *ns*). ERRβ was significantly reduced in the 13-month old DBA/2J mice in contrast to D3, D9-10, and D11-12 groups (Fisher's LSD; *P* < 0.001 in all comparisons; Figure 5B).

### FG density and percent area of intact FG in retina are correlated

Other studies of retrograde transport use FG-positive cell density in the retina as the primary measure of transport (see for example Buckingham et al., 2008). However, to perform within-animal comparisons between retrograde and anterograde transport, we established a variable that allowed direct comparison of FG in the retina with percent area fraction of CTB and ERRβ in the SC. Using montaged image tiles of the entire retinal surface (See Figure 2, bottom row), we quantified the percent area of intact FG label as well as FG-positive cell density in the same retina for all our mice and found that these variables were highly correlated (Pearson's *r* = 0.80, *P* < 0.0001; Figures 5C,D). Therefore, we used percent area intact FG in the retina to compare retrograde label with anterograde and structural labels.

### Retrograde transport (FG) in the retina is slightly reduced at 9-months but is largely intact compared to anterograde transport

In contrast to our pre-glaucomatous D3 mice, the percent area of intact FG was significantly reduced in the D9-10 group (Figure 5C) (Fisher's LSD; *P* = 0.02), D11-12 group (*P* < 0.001), and D13 group (*P* < 0.001). Intact retrograde transport levels were similar between the D9-10 and D11-12 groups (*P* = 0.312, *ns*), but showed a further reduction in the D13 age group (Fisher's LSD; *P* < 0.001 in both sets of comparisons).

**Figure 6:**
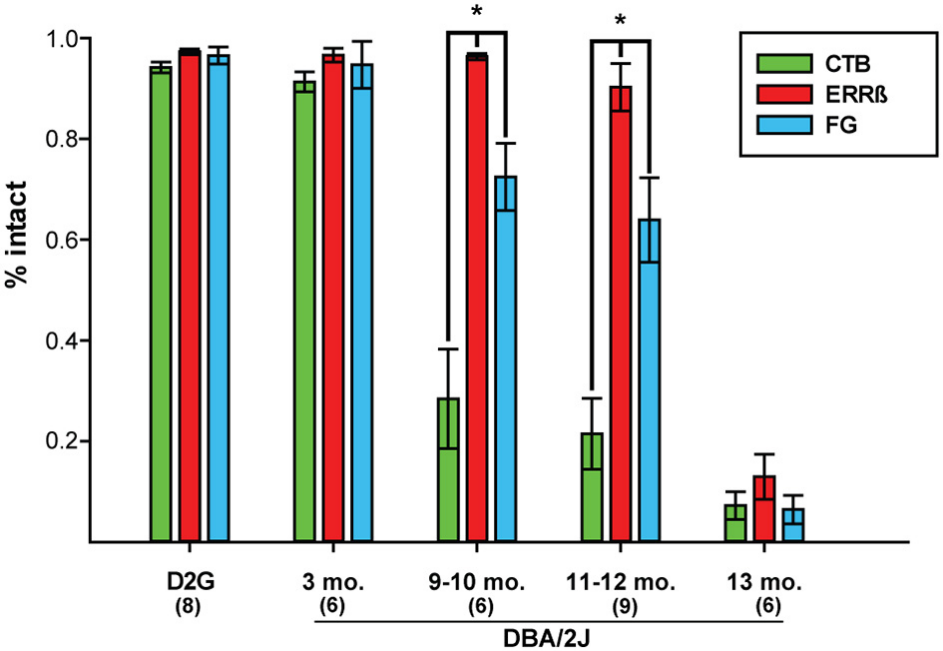
**Mean percent intact CTB (anterograde tracer), ERRβ (axon terminal label), and FluoroGold (FG; retrograde tracer) label shows respective loss of each tracer within age groups**. Within-subjects' statistical analyses showed that there are no differences in the amount of each label in the DBA/2J*-Gpnmb*^+^ (D2G) control and 3-month old (mo.) pre-glaucomatous DBA/2J groups (*Paired t-test*: *P* = *ns* in all cases). Retinal retrograde (FG) label was significantly more intact than collicular anterograde CTB label in glaucomatous DBA/2J at 9–10 mo. (*P* = 0.03) and 11–12 mo. (*P* = 0.02). ERRβ label remained significantly more intact than FG label in DBA/2J mice at 9-10 mo. (*P* = 0.02) and 11–12 mo. (*P* = 0.005). There were no differences in the magnitude of intact label between CTB, ERRβ, and FG by 13-mo (*P* = *ns*). Asterisks and brackets indicate statistically significant differences between specific groups. Number of cases per group are shown in parentheses. Error bars show standard error of the mean for each group.

We used paired *t*-tests to assess within-subject differences in the magnitude of CTB, FG, and ERRβ label for each age group of animals tested. In both the D2G control and D3 groups, there were no measurable differences in the magnitude of label between anterograde, retrograde, and structural markers. However, in the D9-10 group, the amount of intact FG was significantly greater than CTB, *t*_(5)_ = 2.35, *P* = 0.03 (Figure 6). This difference was maintained in the D11-12 group [*t*_(8)_ = 3.059, *P* = 0.02] before dropping to comparable levels in the D13 group.

### Structural integrity of the SC is maintained beyond retrograde transport loss

Normal collicular ERRβ label persisted through 12 months of age as shown in Figure 5B and was significantly greater than retinal FG label in the D9-10 [*t*_(5)_ = −3.41, *P* = 0.02] and D11-12 [*t*_(8)_ = 3.85, *P* = 0.005] groups (Figure 6). Along with the previous data on CTB label magnitude, this finding indicates that the structure of the SC and some components of the RGC projection remained intact after both forms of transport showed significant deficits. In the oldest age group (D13), both forms of transport showed over 90% deficits in intact label with a corresponding 86% reduction in ERRβ. Although structural label was not maximally lost, ERRβ and the transport labels did not differ significantly at D13 (Figure 6).

**Figure 7:**
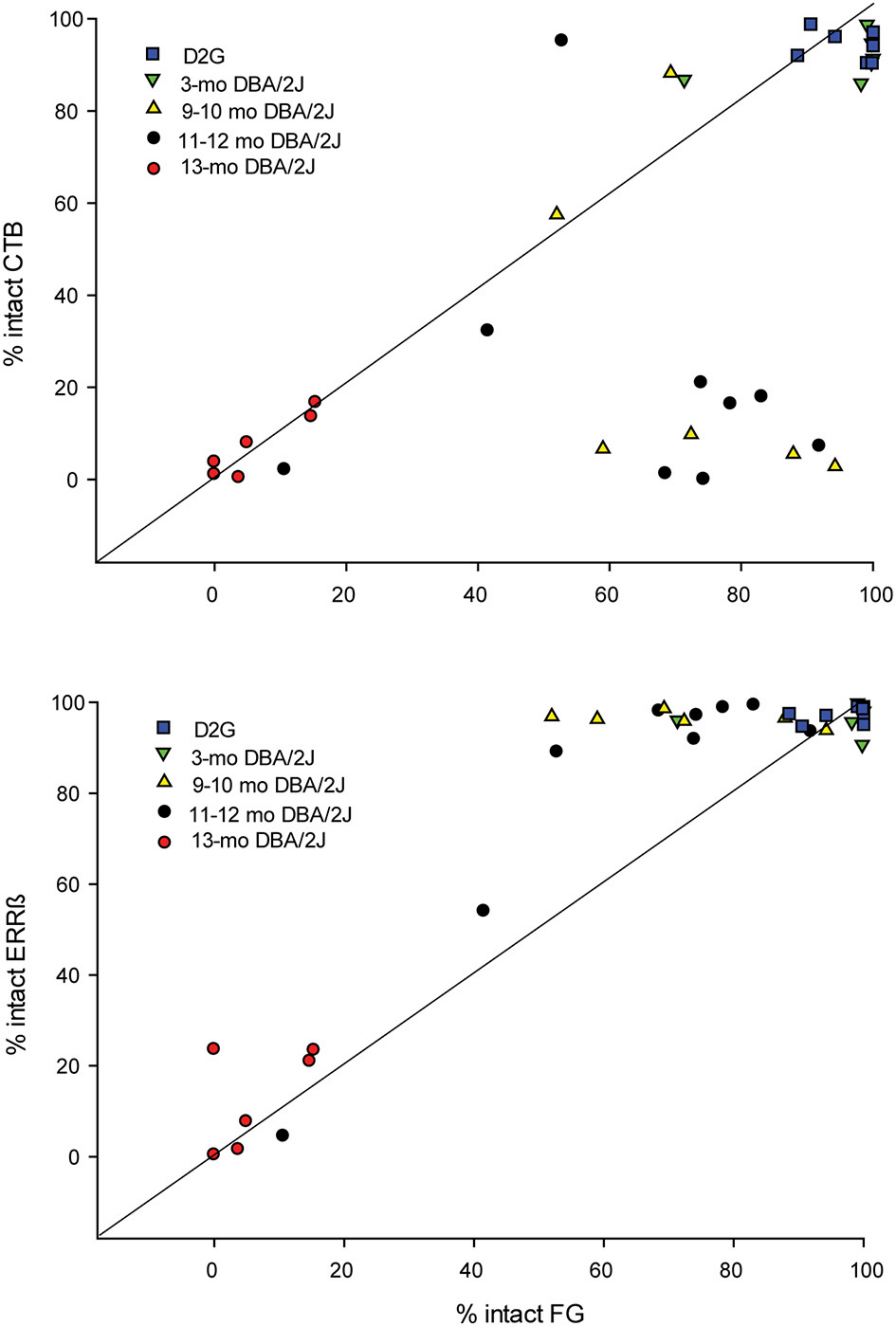
**(A)** Percent area of intact anterograde transport (CTB; y - axis) in the superior colliculus (SC) in comparison with percent area of intact FluoroGold (FG; x - axis) in the retina for individual projections. We have plotted a unity line (slope = 1) where points would be expected to fall if the percent of intact anterograde and retrograde transport changed similarly across age groups. Only the fully intact (DBA/2J*-Gpnmb*^+^ (D2G) and 3-mo. DBA/2J) and extremely pathological (13-mo. DBA/2J) cases fall along this line. The majority of 9–10 mo. old and 11–12 mo. old DBA/2J cases fall well below the unity line, indicating that CTB transport is diminished across these age groups while retrograde remains intact. **(B)** Percent area of intact axon terminals/structure (ERRβ; y-axis) in the SC vs. percent area of intact retrograde transport (FG; x - axis) in the retina for individual projections. We have plotted a unity line (slope = 1) where points would be expected to fall if percent of intact retrograde (FG) transport in the retina and structural integrity of the superior colliculus (ERRβ) changed similarly across age groups. In contrast to CTB data, we find that the majority of 9–12 mo. DBA/2J cases fall above the unity line, indicating that ERRβ remains intact across more ages than does FG.

Differential reductions in transport by individual projections are summarized in Figure 7. The top plot compares anterograde (CTB) and retrograde (FG) transport in the same projection. We have plotted a unity line where we would expect the points to fall if both forms of transport were similarly affected. Supporting our group observations, we have a significant cluster of cases below the unity line, indicating that we saw much larger anterograde deficits than retrograde deficits at younger ages. As shown in the bottom plot of Figure 7, when we compared retrograde transport to our RGC projection structural marker in the SC (ERRβ) we found a significant cluster of cases above the unity line, indicating FG transport deficits precede loss of ERRβ immunoreactivity in the SC.

## Discussion

Both anterograde and retrograde transport have not been previously assessed within the same projection—primarily due to technical limitations of the surgeries used for FG application. Historically, FG application required one or more large injections into the SC followed by intracranial insertion of FG-soaked pledgets for additional tracer exposure (see Sellés-Navarro et al., 1996; Villegas-Pérez et al., 1996). This method resulted in extensive damage to the SC, precluding analysis of the RGC projection, anterograde transport levels, or FG exposure in this structure (Buckingham et al., 2008; Soto et al., 2008). Therefore, we modified the number, size, and approach of our intracranial injections to achieve complete uptake and transport to the retina while preserving the structure of the SC for immunohistochemical analysis.

Using tract tracing of the central visual pathway within the same projection, we found that severe deficits in anterograde transport appeared at an earlier age than deficits in retrograde transport (Figures 5, 6). Anterograde transport is reduced by almost 70% at 9–10 months of age which is comparable to (and statistically indistinguishable from) the near-maximal 92% loss in label shown at 13-months. This is a 3-fold greater reduction in label than was seen with retrograde transport, which, at 9–10 months was only reduced by 23%. Additionally, retrograde transport remained largely intact until 13-months where it then dropped off to values comparable to anterograde transport—a finding consistent with other published reports that assessed retrograde transport in DBA/2J (Buckingham et al., 2008). This addresses one of the most important open questions raised by previous studies examining these subjects (Buckingham et al., 2008; Crish et al., 2010). Together these data indicate that the RGC axonal projection remains at least semi-functional after anterograde transport loss in glaucoma.

Our results are consistent with other studies that have examined these forms of transport separately in rodent glaucoma models. In the DBA/2J mouse, anterograde transport deficits have been shown at 8–10 months of age with pervasive loss by 12-months while RGC axons and presynaptic terminals in the SC can persist up to 18 months (Crish et al., 2010; Dapper et al., 2013). Retrograde transport deficits have been observed to occur later around 13 months (Buckingham et al., 2008; Salinas-Navarro et al., 2009b; Vidal-Sanz et al., 2012). These findings address a second major question from these original studies, indicating that retrograde transport loss occurs before overt loss of structure in the projection. This suggests previously postulated mechanisms such as intra-axonal cytoskeletal abnormalities, motor protein issues, or metabolic deficits likely play a large role in the transport deficits seen in this model (Baltan et al., 2010; Crish et al., 2010; Crish and Calkins, 2011). Collectively, these findings indicate that axonal transport deficits are pre-degenerative and may be differentially impaired in glaucoma. This data is consistent with results found in other neurodegenerative conditions (Lafuente Lopez-Herrera et al., 2002; Avilés-Trigueros et al., 2003; Morfini et al., 2009b; Crish and Calkins, 2011).

We assessed the percent area of intact retina by measuring the areas of absent FG and subtracting this from the overall retinal area. We found this variable to be highly correlated with density of FG-positive RGCs—however, it is important to note this percent-intact variable fails to take into account any possible reductions in the density of those intact areas. It is possible that subtle reductions in RGC density of those “intact” areas could show further deficits in our studies. However, given the sectorial nature of the deficits seen in glaucoma (Jakobs et al., 2005), and that the DBA/2J mice we analyzed were of ages representing high likelihood of pathology, it is unlikely these subtle differences would have influenced our results.

### Transport function in glaucoma

Impaired movement of cargo along the axon initiates several degenerative mechanisms including distal Wallerian degeneration and apoptosis due to neurotrophin deprivation (see Coleman, 2005). There are a large number of possibilities that can negatively affect proper transport function, including modification of molecular motors, cytoskeletal disruption, and metabolic dysfunction (Coleman, 2005; Shea and Chan, 2008; Morfini et al., 2009a; Crish and Calkins, 2011). These changes can often affect very specific parts of transport such as different compartments of the axon or the direction of cargo movement away from the cell body or toward the cell body (Shea and Beaty, 2007). Due to the complexity of the nervous system, most of the evidence reporting differential transport effects in pathology are *ex vivo* (see for example, Brady et al., 1990; Morfini et al., 2007; Motil et al., 2007; Lee et al., 2011; LaPointe et al., 2013).

Differential transport loss in glaucoma may be due to greater sensitivity of anterograde transport to an intra-axonal blockade (Wang et al., 1995; Ross et al., 2006; see discussion in Shea and Beaty, 2007). These intra-axonal blockades may result from the disorganization and breakdown of the cytoskeleton with the consequential accumulation of protein cargo (Shea et al., 2004; Coleman, 2005; Crish et al., 2010). Dynein, the molecular motor that mediates retrograde transport, has greater lateral and bidirectional movement along microtubules within the axon. Therefore, dynein may more easily navigate around these blockades by translocating to other, intact microtubule tracks at that location. In contrast, the molecular motor that drives anterograde transport, kinesin, does not have this capability, therefore making it vulnerable to axonal blockade (Wang et al., 1995; Ross et al., 2006).

Additionally, the differential effects we have shown on axonal transport may result from regulation of the molecular motors themselves. Kinesin can be dysregulated or inhibited by kinases such as GSK-3β, p38, and JNK, and activity of these kinases is notably increased in neurodegenerative disorders (see Brady and Morfini, 2010; Wang et al., 2014 for discussions). Notably, p38 has recently been implicated in axonal transport deficits and distal axonopathy in DBA/2J mice (Dapper et al., 2013), JNK is rapidly upregulated after RGC injury (Fernandes et al., 2014), and investigations into GSK3β signaling pathways are ongoing in our laboratories.

A common hypothesis in glaucoma is that axonal transport is blocked by mechanical compression of the optic nerve head (ONH) where the RGC axons leave the eye. If this were the case then we would expect anterograde and retrograde transport to be similarly affected, but here in the DBA/2J mouse they are not. Furthermore, our observations of reduced levels of β-Tubulin and increased levels of pNF support the idea that axon transport is blocked by intra-axonal changes far removed from the ONH. Both microtubule breakdown and abnormal phosphorylation of neurofilaments have profound effects on axonal transport and changes to both these proteins may play a large role in the deficits seen here (Shea and Beaty, 2007). These intra-axonal changes may also result from disruptions in kinase signaling pathways (Wang et al., 2014). Even in extreme cases of CTB and ERRβ loss in the SC, CTB-positive axons, varicosities, or end bulbs are evident in the optic nerve and tract as shown in Figure 4 as well as in previous studies (Crish et al., 2010). This indicates that these axons (even one undergoing distal axonopathy as shown in Figure 4, bottom row) are still connected to their cell bodies in the retina. It is important to note that this idea does not detract from the role of the ONH in the development and progression of glaucoma—critical observations regarding the onset of pathological change have been noted in the ONH and the retina—and pathology in neurons (e.g., blocked axon transport and axonopathy in this region) is often far-removed from the site of the stressor (Conforti et al., 2007; Cuchillo-Ibanez et al., 2008).

### Structural persistence of the SC

Structural persistence of the RGC projection to the SC (as measured by intact ERRβ) was maintained in glaucomatous mice from 9 to 12 months of age and significant decreases in ERRβ label were only first measured at 13-months of age. Contrasting with both anterograde and retrograde labels that first showed significant reductions in the 9–10 month age group, our findings suggest that transport deficits are likely due to physiological or functional abnormalities as opposed to overt structural loss. Our results did differ from previous reports of Crish et al. (2010) in that our ERRβ label for intact SC was significantly reduced at 13-months, whereas significant reductions in ERRβ did not appear in those studies until 15–22 months. However, there is a significant amount of variability in structural persistence beyond 12 months of age (Crish et al., 2010) and it is possible that our smaller sample size of dual-traced animals captured more extreme deficit in ERRβ at 13-months. However, this does not affect our overall conclusions.

### Cytoskeletal alterations, neuronal signaling, and transport deficits

Breakdown or modification of cytoskeletal elements or motor proteins are common pathologies in neurological disorders such as Alzheimer's Disease and amyotrophic lateral sclerosis and can negatively affect neuron structure and function in terms of cellular integrity, axonal transport, and signaling (Coleman, 2005; Conforti et al., 2007; Cuchillo-Ibanez et al., 2008; Shea and Chan, 2008; Morfini et al., 2009a; Shea et al., 2009). It is possible that disruptions in axonal transport shown in our current studies result from intra-axonal blockades caused by aberrant cytoskeletal organization/breakdown or dysregulation of molecular motors that may reflect changes occurring in glaucoma occurring before overt degeneration of axons or cell bodies. A major question this research raises is what is the connection between these changes and other early pathologies seen in glaucoma? While anterograde transport interruptions can drive axonal degeneration distal to the blockage (see Coleman, 2005), the relationship between these deficits and early dendritic remodeling in the retina (Jakobs et al., 2005; Della Santina et al., 2013), inflammation (Bosco et al., 2011), and physiological deficits in RGC activity (Della Santina et al., 2013; see Saleh et al., 2007 for DBA/2J; Banitt et al., 2013 for humans) or action potential conduction (Baltan et al., 2010) remain to be determined. Inflammation as measured by increased microglial number and activation occurs quite early in the DBA/2J inner retina and ONH, peaking by 3 months (Bosco et al., 2011). Since microglial release of NO can halt anterograde axon transport (Stagi et al., 2005), there may be a negative impact of these cells on axon transport quite early, though undetected at 3 months in our study. The significant decreases in pattern electroretinogram (PERG) amplitude that affect the DBA/2J by 6 months of age (Saleh et al., 2007) suggest changes in inner retina activity that may be carried over into optic nerve as decreases in compound action potential amplitude, an observation made in DBA/2J mice with high IOP by 6 months of age (Baltan et al., 2010). Alternatively, decreases in axon transport of mitochondria in the DBA/2J optic nerve, for example, could also explain loss of compound action potential amplitude. Our study focused on transport changes very early (3 months) and late (9 months and beyond), so additional analysis of anterograde axon transport in between may be able to distinguish among the competing mechanisms for the transport pathology observed. Such information may provide exciting new avenues for treatments targeted at restoring function and substructure in glaucomatous projections that still remain largely intact.

## Author contributions

Christine M. Dengler-Crish and Matthew A. Smith contributed equally to this paper and were involved in the design, acquisition, analysis and interpretation of data as well as writing and revising the manuscript. Samuel D. Crish and Denise M. Inman conceptualized and designed the studies; analyzed and interpreted the data and assisted in writing and critically revising the manuscript. Jesse W. Young assisted in the critical revision of the manuscript; designed and coded the ImageJ macro in order to analyze and interpret neuroanatomical data. Gina N. Wilson was involved in the acquisition and analysis of data and assisted in revisions of the work.

### Conflict of interest statement

The authors declare that the research was conducted in the absence of any commercial or financial relationships that could be construed as a potential conflict of interest.
